# A potential role for inflammatory cytokines in a rare late-onset capsular block syndrome: a case report

**DOI:** 10.1186/s12886-024-03320-0

**Published:** 2024-02-02

**Authors:** Ying-Hua Du, Xiao-Fang Liang, Kazuyuki Hirooka, Hui-Ka Xia, Zhi-Yang Jia

**Affiliations:** 1 Department of Ophthalmology, Beijing TianTan Hospital, Capital Medical University, No. 119 South Fourth Ring West Road, Fengtai District, 100070 Beijing, China; 2 Department of Ophthalmology, Hiroshima University Hospital (Medical), Hiroshima, Japan; 3 Department of Ophthalmology, Hebei General Hospital, 050051 Shijiazhuang, China

**Keywords:** Case report, Capsular block syndrome, Late-onset, Cataract surgery, Inflammatory cytokines

## Abstract

**Background:**

Late-onset capsule block syndrome (CBS) is a rare complication of cataract phacoemulsification and the implantation of a posterior chamber intraocular lens (PCIOL), which manifests six months to years after surgery. The hallmark of CBS is the formation of an opaque liquid substance between the implanted intraocular lens (IOL) and the posterior capsule. However, its pathogenesis remains unclear.

**Case presentation:**

A 64-year-old female patient with chronic angle-closure glaucoma (axis length < 21 mm) underwent trabeculectomy surgery combined with phacoemulsification and PCIOL. After a 4-year follow-up, a decline in visual acuity occurred in her right eye due to the location of opaque fluid in the visual axis and distension of the capsular bag. The initial course of action was to release the trapped fluid. Neodymium: yttrium-aluminum-garnet (Nd: YAG) laser capsulotomy could not be employed due to her non-dilating pupil and high extension of the posterior capsule. Subsequently, anterior capsule peeling and anterior segment vitrectomy surgery were performed. The depth of the anterior chamber (ACD), the distance between the face of the retro-IOL and the posterior capsule, the best-corrected visual acuity (BCVA), and the visual quality (VQ) were measured both before and after surgery. Inflammatory cytokine levels in the opaque substances (OS) trapped between the PCIOL and the posterior capsule were assessed using a flow cytometer and compared to normal statistical data in aqueous humor. After surgery, the patient experienced a significant improvement in BCVA and VQ. The distance between the face of the retro-IOL and the posterior capsule was on the verge of disappearing. However, ACD did not differ between pre- and post-operatively. Interleukin-8 (IL-8) and basic fibroblast growth factor (BFGF) concentrations were higher in the OS than in aqueous humor, especially in the former. However, the concentration of vascular cell adhesion molecule (VCAM) in the OS was lower than in aqueous humor.

**Conclusions:**

Anterior segment vitrectomy surgery proved to be a successful treatment for late-onset CBS, presenting a challenging case. In the human lens, inflammatory cytokines originating from the opaque substances may contribute to abnormal metabolism in the sealed area, a consequence of late-onset CBS.

## Background

Late-onset capsular block syndrome (CBS) is an infrequent complication arising from continuous curvilinear capsulorhexis (CCC) that typically becomes apparent six months to several years following cataract surgery with posterior chamber intraocular lens (PCIOL) implantation [1–3]. Phacoemulsification has been reported to be associated with CBS in approximately 0.73–1.0% of cases [4]. The description by Miyake et al. highlights late-onset CBS as characterized by the accumulation of opaque substances (OS) composed of residual lens epithelial cells [5]. Miyake et al. further classified CBS into early postoperative, induced by elevated irrigation pressure during hydro dissection procedures, and intraoperative CBS, resulting from osmotic gradient accumulation within two weeks post-operation. Clinical manifestations of early postoperative CBS include capsular dilation, IOL displacement, anterior chamber shallowing, and an unexpected myopia shift [6]. In contrast, late-onset CBS does not exhibit these features, and its identification may be delayed until the best-corrected visual acuity (BCVA) has gradually declined to a certain extent [7].

The pathophysiology of late-onset CBS encompasses various perspectives. While lens epithelial cells, when present, occasionally yield insights into the composition and source of the OS responsible for the relatively high alpha-crystallin and low albumin levels observed by Eifrig and Bao [8, 9], an alternative perspective considers the potential for passive invasion by *Propionibacterium acnes* [10, 11]. However, no further evidence substantiates the existence of *P. acnes*-associated endophthalmitis during fluid release following Nd: YAG capsulotomy or other therapies [12–14]. Huang’s findings indicate significantly higher levels of interleukin-1 and tumor necrosis factor-alpha in the OS compared to aqueous humor [15]. Nevertheless, insufficient data hinders the establishment of a connection between the opaque material in rare late-onset CBS and inflammatory cytokines.

Neodymium: yttrium-aluminum-garnet (Nd: YAG) laser capsulotomy has traditionally been the standard treatment for late-onset CBS due to its simplicity and minimal intrusion. However, in our case, the pupil could not be dilated, the OS was dense, and the posterior capsule showed high extension. Consequently, laser capsulotomy could not effectively target the desired area. Therefore, it became necessary to explore and implement a surgical intervention.

We aimed to highlight the characteristics of late-onset CBS in this study, evaluate various surgical treatments, and investigate the role of inflammatory cytokine levels in the etiology of late-onset CBS.

## Case presentation

A 64-year-old female patient presented with blurry, double, and starburst vision in her right eye, accompanied by a one-year history of decreasing visual acuity. Her ocular background involved chronic angle-closure glaucoma. Four years prior, she underwent trabeculectomy surgery along with cataract phacoemulsification and PCIOL implantation in both eyes. Additionally, she has a medical history of osteoarthritis and an inguinal hernia. Two months post-surgery, the anterior capsule of her left eye contracted rapidly, and Nd: YAG capsulotomy therapy was employed to restore visual acuity. Throughout the follow-up, intraocular pressure in both eyes ranged between 18 and 21 mmHg.

**Fig. 1:**
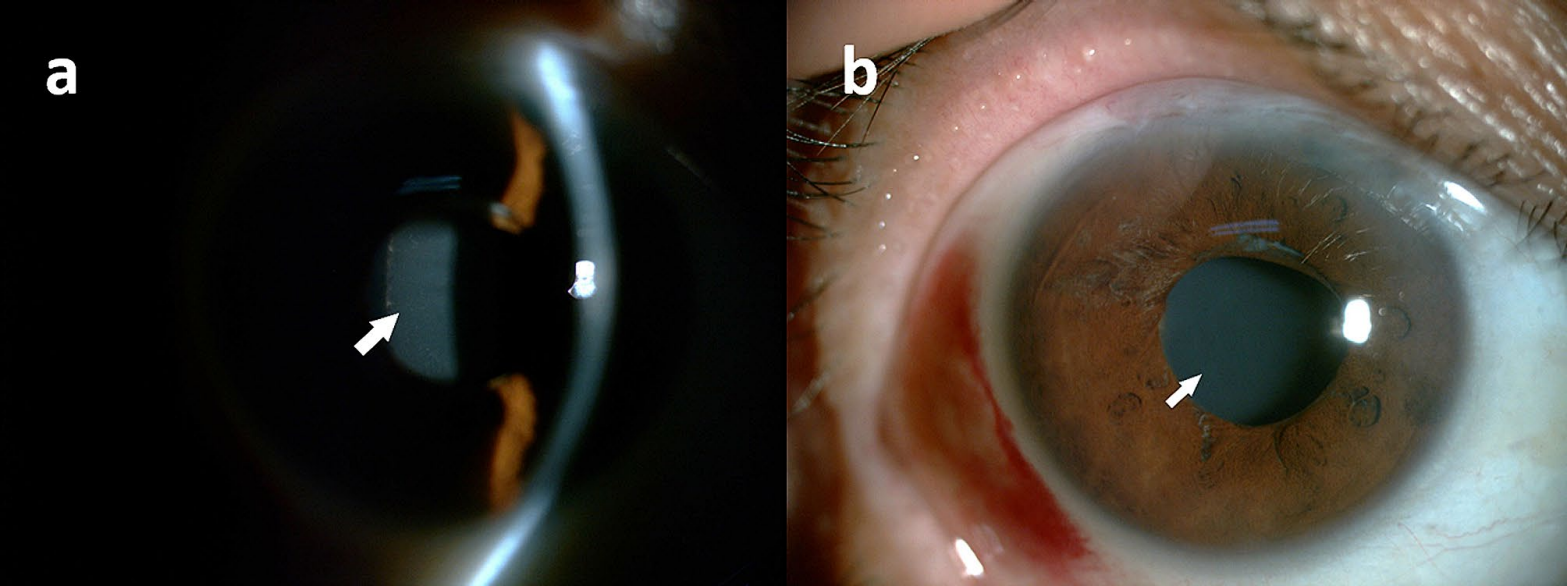
A slit **(a)** and diffuse beam **(b)** lamp biomicroscopic examination revealed some turbid, milky-white fluid (white arrow) behind the retro-IOL region with a non-dilated right pupil

**Fig. 2:**
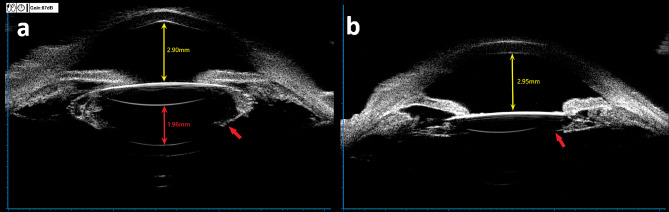
**(a)** Preoperative biometry using ultrasonic biomicroscopy shows an anterior chamber depth of 2.90 mm (yellow line) and a distance between the artificial lens and posterior capsule of 1.96 mm (red line) (UBM, SW-3200 L, Tianjin). A mild echo was reflected following the IOL and posterior capsule, with a dot/cluster echo (red arrow) near the bottom of the hyper-distension posterior capsule. The UBM revealed no space between the posterior iris surface and the interface of the lens complex. **(b)** Six months following surgery, biometry showed an anterior chamber depth of 2.95 mm (yellow line), the posterior lenticular capsule rim (red arrow), and no aberrant deposits after IOL as indicated by UBM

Recently, her right eye’s best-corrected visual acuity (BCVA) had been gradually declining and was now 6/20. Clear corneas, typical anterior chambers devoid of cells or flare, and a properly positioned posterior chamber intraocular lens (PCIOL) were observed during slit lamp inspection. However, the pupil was only slightly dilated at 3.5 mm. Examination revealed collections of the OS in the right eye trapped between the retro-IOL surface and the hyper-distended posterior capsule in the posterior chamber (Fig. 1). Utilizing the IOL master (Carl Zeiss Meditec AG, Germany) and ultrasound biomicroscopy (UBM, SW-3200 L, Tianjin), preoperative biometry measured an axial length of 20.66 mm, anterior chamber depth (ACD) of 2.90 mm, and the distance between the PCIOL surface and posterior capsule of 1.96 mm, respectively (Fig. 2). No space was observed between the posterior iris surface and the lens-complex interface. According to UBM, a weak echo was reflected following the IOL and posterior capsule, with a dot/cluster echo towards the posterior capsule’s bottom. B-scan ultrasound (Esaote, Genova, Italy) did not reveal any noteworthy abnormalities. Simultaneously, measurements for the left eye indicated a 20/20 BCVA, a 20.31 mm axial length, and a 2.81 mm ACD. Examination of the posterior segments of both eyes revealed nearly normal macular anatomy, with a cup-to-disc ratio of 0.5 in both eyes. Itrace’s visual quality (VQ) analysis indicated that coma and trefoil in the internal eye were the primary causes of blur, double vision, starbursts, and decreased contrast sensitivity. The presence of a trapped OS and a hyper-distended posterior capsule could contribute to confusing low VQ across all indications. All evidence suggested late-onset CBS occurring in her right eye based on the trapped OS and the apparent absence of anterior and/or posterior inflammation. However, Nd: YAG laser capsulotomy therapy was constrained by the small pupil and hyper-distended posterior capsule.

Once permission was granted, the patient underwent local anesthesia for pars plana anterior vitrectomy, pupil reformation, posterior capsulotomy, release of opaque material with an inflammatory cytokine test, and other necessary procedures. Iris hooks assisted in visualizing a roughly 3.5 mm-sized white anterior capsule, though shrinking during surgery, which showed signs of fibrosis around its entire perimeter. No retained cortex was observed, and the posterior capsule remained non-turbid. Using a 23-gauge trocar, a 3.5 mm pars plana port cannula was created from the limbus. Initially, attempts to remove OS from the posterior chamber using a 30-gauge needle were hindered by negative pressure, indicating a sealed area between the posterior capsule and the retro-IOL surface. Subsequently, the posterior capsule was penetrated, and the capsular bag contents were aspirated by a cutter through the super-temporal port, with irrigation closed infra-temporally. For the analysis of inflammatory cytokines, the aspirated fluid was promptly transferred and maintained at a temperature of 4 °C. The remaining anterior capsular, enlarged to a diameter of 6 mm, was released after careful peeling off of the fibrosis anterior capsular ring. The ring material was then fixed for pathology study. A 4 mm diameter posterior capsulotomy was performed in the center using a 23-gauge vitrectomy, followed by a local anterior vitrectomy. Pathological examination of the 3 × 2 mm area and 1 mm thick fibrosis anterior capsular material revealed innocent fibrotic cystic tissue with minor pigment accumulation. Cytokine analysis using a flow cytometer multiple array assay device identified changes in some cytokines, including up-regulation of BFGF, IL-8, and down-regulation of VCAM (Table 1).

**Table 1:** In the fluid sample from late-onset CBS compared to that from aqueous water, certain inflammatory cytokines had changed

Order	Test items	Result	Unit	Reference range
1	IL-10/IL-6	0.08		< 1.0
2	VEGF	11.4	pg/ml	0 ~ 40.0
3	BFGF	31.3↑	pg/ml	< 1.0
4	IL-6	1.2	pg/ml	1.0 ~ 50.0
5	IL-10	0.1	pg/ml	0 ~ 5.0
6	VCAM	21.4↓	pg/ml	200 ~ 1000
7	IL-8	37.3↑	pg/ml	0 ~ 20.0

**Fig. 3:**
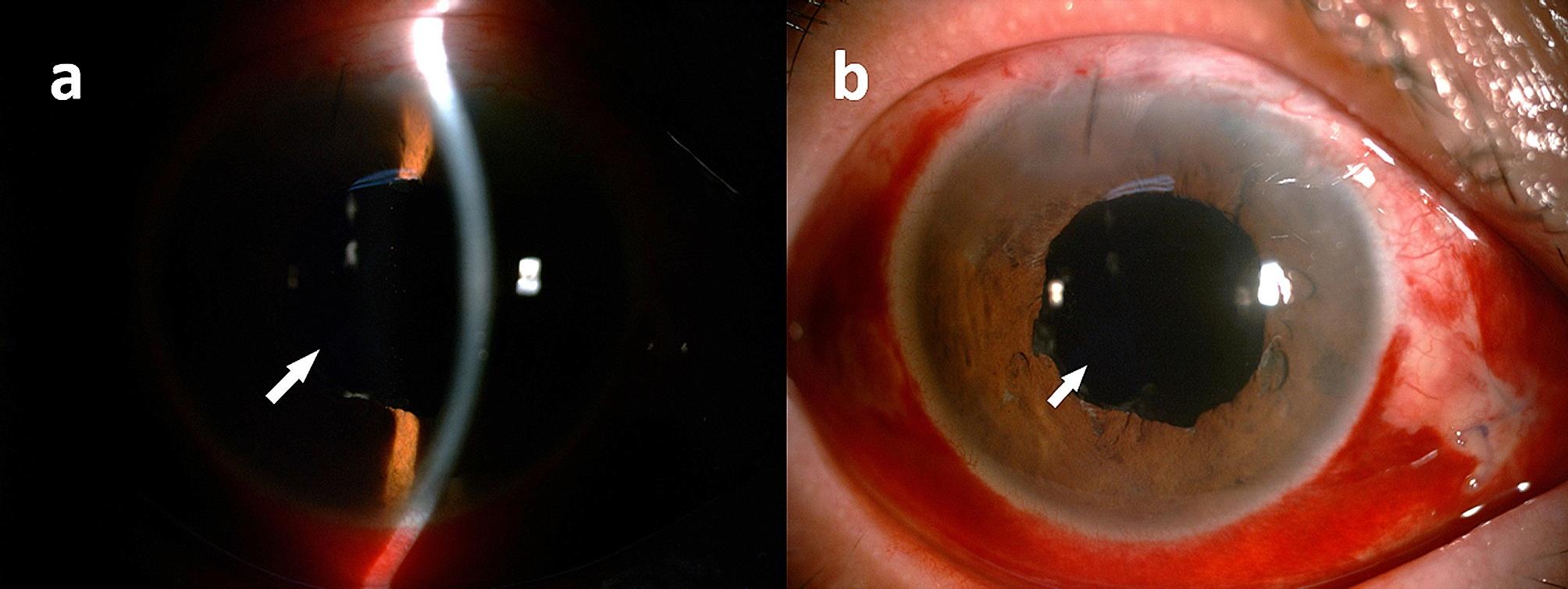
Post-operation of 3 days, a slit lamp biomicroscopic examination by slit **(a)** and diffuse beam **(b)** indicated no fluid (white arrow) behind the retro-IOL space

The patient was monitored for 6 months, with a positive postoperative phase and an improvement in BCVA to 20/25. The anterior chamber’s depth increased slightly from 2.90 to 2.95 mm. This procedure also addressed other CBS issues, such as improved pupil function, release of the trapped OS, proper placement of the IOL in the bag, and the absence of aberrant IOL deposits (Fig. 3). The absence of visual complaints confirmed the successful treatment of coma and trefoil in the internal eye through Itrace analysis. Due to a history of glaucoma, fundoscopy and optic coherence tomography revealed moderate optic atrophy.

## Discussion and conclusions

Few examples of CBS have been documented in the literature. Vision loss was primarily described in previously published cases, with the typical myopic shift caused by the IOL moving forward and a shallow anterior chamber. However, our late-onset case did not exhibit these signs and had a fairly normal anterior chamber, deviating from Zhu’s case report, which showed a clear difference in the anterior chamber range of 0.20 to 0.45 mm [16].

In our case, the main causes of visual complaints, such as internal coma and trefoil, were the trapped OS in the visual axis and the distension of the capsular bag. What about the OS resources? A report explained that metaplastic IECs might secrete opaque material gathered in the retro-lenticular fluid [5]. Due to challenges in gathering these components during the therapeutic course, the composition of the trapped opaque fluid became unclear. Considering the sealed space as a newborn, inflammatory cytokines should not be disregarded during surgery recovery. To our knowledge, no literature has explored their role in the metabolism of the trapped opaque fluid. In our study, we discovered that BFGF and IL-8 had increased expression when comparing the aspirated fluid contents with aqueous water using flow cytometry, particularly the former. VCAM expression was downregulated concurrently. VEGF, IL-6, and IL-10 concentrations were comparable to those seen in aqueous water. BFGF, a key secretory signaling protein expressed in practically every tissue, played a role in cell proliferation, regeneration, migration, and survival in both normal and pathological situations [17]. Its increased expression suggested that late-onset CBS was in a relatively positive condition. Additionally, BFGF was used to boost IL-8 expression in our case, similar to Kim’s case [18]. IL-8, an interleukin, characterized inflammation and host defense [19]. In our review of late-onset CBS, there was a small elevation of IL-8 expression, along with localized weak reactivity. Furthermore, the potential underlying mechanism linked to VCAM downregulation in our case was the lack of a scaffold or trigger for leukocyte migration [20]. The increase in BFGF in response to host defense circumstances and the local response to IL-8/VCAM, resulting in late-onset CBS, were just two examples of the inflammatory cytokines in our study indicating abnormal metabolism in the sealed posterior chamber area.

Based on our knowledge, we hypothesize that a minor inflammatory cytokine could act as a self-defense mechanism during cataract surgery, where CCC is a crucial condition for forming a sealed compartment. As the anterior capsule grows and makes tighter contact with the artificial lens, a sealed and isolated space emerges between the PCIOL and the posterior capsule. This unusual microenvironment leads to the accumulation of fluid containing cellular components such as BEGF, IL-8, and VCAM. These cellular factors influence both the underlying cause and the resolution of the issue.

## Data Availability

The corresponding author, Ying-Hua Du, MD, PhD, has full control of all primary data contributed to guide or conduct the experiment and agrees to allow the Journal of BMC ophthalmology to review their data upon request.

## Abbreviations

CBS: capsule block syndrome
ACD: the depth of the anterior chamber
IOL: implanted intraocular lens
BCVA: the best-corrected visual acuity
VQ: the visual quality
OS: the opaque substances
IL-8: Interleukin-8
BFGF: basic fibroblast growth factor
VCAM: vascular cell adhesion molecule
CCC: continuous curvilinear capsulorhexis
PCIOL: posterior chamber IOL implantation
UBM: ultrasound biomicroscopy
